# Retracted: Isolation of a Reassortant H1N2 Swine Flu Strain of Type “Swine-Human-Avian” and Its Genetic Variability Analysis

**DOI:** 10.1155/2022/9860961

**Published:** 2022-03-18

**Authors:** BioMed Research International

BioMed Research International has retracted the article titled “Isolation of a Reassortant H1N2 Swine Flu Strain of Type “Swine-Human-Avian” and Its Genetic Variability Analysis” [1], due to concern with the figures.

In Figure 2, The electron microscopy image of H1N2 Subtype Swine Influenza Virus was found to be an image of the H3N2 subtype swine influenza virus which had been published by the authors in the *Chinese Journal of Preventive Veterinary Medicine* [2].

Based on this concern, the article is being retracted with the agreement of the editorial board.

The authors agree to the retraction and the notice.
